# Using Culture Sensitivity Reports to Optimize Antimicrobial Therapy: Findings and Implications of Antimicrobial Stewardship Activity in a Hospital in Pakistan

**DOI:** 10.3390/medicina59071237

**Published:** 2023-07-02

**Authors:** Ummara Altaf, Zikria Saleem, Muhammad Furqan Akhtar, Waleed Mohammad Altowayan, Abdulmajeed A. Alqasoumi, Mohammed Salem Alshammari, Abdul Haseeb, Fahad Raees, Mohammad Tarique Imam, Narjis Batool, Muhammad Masoom Akhtar, Brian Godman

**Affiliations:** 1 Riphah Institute of Pharmaceutical Sciences, Riphah International University, Lahore 54000, Pakistan; ummara.altaf1994@gmail.com (U.A.); mfurqan.akhtar@riphah.edu.pk (M.F.A.); 2 Department of Pharmaceutical Services, Ghurki Trust Teaching Hospital, Lahore 54000, Pakistan; 3 Department of Pharmacy Practice, Faculty of Pharmacy, Bahauddin Zakariya University, Multan 60800, Pakistan; 4 Department of Pharmacy Practice, College of Pharmacy, Qassim University, Buraydah 52571, Saudi Arabia; w.altowayan@qu.edu.sa (W.M.A.); a.alqasomi@qu.edu.sa (A.A.A.); 5 Department of Pharmacy Practice, Unaizah College of Pharmacy, Qassim University, Unaizah 56215, Saudi Arabia; m.alshammari@qu.edu.sa; 6 Department of Clinical Pharmacy, College of Pharmacy, Umm Al-Qura University, Makkah 24382, Saudi Arabia; amhaseeb@uqu.edu.sa; 7 Department of Medical Microbiology, Faculty of Medicine, Umm Al-Qura University, Makkah 24382, Saudi Arabia; frahmed@uqu.edu.sa; 8 Department of Clinical Pharmacy, College of Pharmacy, Prince Sattam Bin Abdulaziz University, Al Kharj 11942, Saudi Arabia; m.imam@psau.edu.sa; 9 Center of Health Systems and Safety Research, Faculty of Medicine, Health and Human Sciences, Australian Institute of Health Innovation, Macquarie University, Sydney 2109, Australia; narjis.batool@stdents.mq.edu.au; 10 Faculty of Pharmacy, Hamdard University Islamabad Campus, Islamabad 700081, Pakistan; m.masoom@hamdard.edu.pk; 11 Strathclyde Institute of Pharmacy and Biomedical Sciences, Strathclyde University, Glasgow G4 0RE, UK; brian.godman@strath.ac.uk; 12 Department of Public Health Pharmacy and Management, School of Pharmacy, Sefako Makgatho Health Sciences University, Pretoria 0208, South Africa; brian.godman@smu.ac.za; 13 Centre of Medical and Bio-Allied Health Sciences Research, Ajman University, Ajman 346, United Arab Emirates

**Keywords:** anti-microbial resistance, anti-microbial stewardship, culture sensitivity reports, costs, definitive treatment, empiric treatment, hospitals, Pakistan

## Abstract

*Background***:** There are concerns with inappropriate prescribing of antibiotics in hospitals especially broad spectrum in Pakistan and the subsequent impact on antimicrobial resistance rates. One recognized way to reduce inappropriate prescribing is for empiric therapy to be adjusted according to the result of culture sensitivity reports. *Objective:* Using culture sensitivity reports to optimize antibiotic prescribing in a teaching hospital in Pakistan. *Methods:* A retrospective observational study was undertaken in Ghurki Trust Teaching Hospital. A total of 465 positive cultures were taken from patients during the study period (May 2018 and December 2018). The results of pathogen identification and susceptibility testing from patient-infected sites were assessed. Additional data was collected from the patient’s medical file. This included demographic data, sample type, causative microbe, antimicrobial treatment, and whether empiric or definitive treatment as well as medicine costs. Antimicrobial data was assessed using World Health Organization’s Defined Daily Dose methodology. *Results:* A total of 497 isolates were detected from the 465 patient samples as 32 patients had polymicrobes, which included 309 g-negative rods and 188 g-positive cocci. Out of 497 isolates, the most common Gram-positive pathogen isolated was *Staphylococcus aureus* (Methicillin-sensitive *Staphylococcus aureus*) (125) (25.1%) and the most common Gram-negative pathogen was *Escherichia coli* (140) (28.1%). Most of the gram-negative isolates were found to be resistant to ampicillin and co-amoxiclav. Most of the *Acinetobacter baumannii* isolates were resistant to carbapenems. Gram-positive bacteria showed the maximum sensitivity to linezolid and vancomycin. The most widely used antibiotics for empiric therapy were cefoperazone plus sulbactam, ceftriaxone, amikacin, vancomycin, and metronidazole whereas high use of linezolid, clindamycin, meropenem, and piperacillin + tazobactam was seen in definitive treatment. Empiric therapy was adjusted in 220 (71.1%) cases of Gram-negative infections and 134 (71.2%) cases of Gram-positive infections. Compared with empiric therapy, there was a 13.8% reduction in the number of antibiotics in definitive treatment. The average cost of antibiotics in definitive treatment was less than seen with empiric treatment (8.2%) and the length of hospitalization also decreased. *Conclusions:* Culture sensitivity reports helped reduced antibiotic utilization and costs as well as helped select the most appropriate treatment. We also found an urgent need for implementing antimicrobial stewardship programs in hospitals and the development of hospital antibiotic guidelines to reduce unnecessary prescribing of broad-spectrum antibiotics.

## 1. Introduction

The emergence of antimicrobial resistance (AMR) is a worldwide problem impacting morbidity, mortality, and costs [1,2]. The irrational use of broad-spectrum antibiotics, particularly for acute respiratory tract infections, is the most common cause of AMR [3]. Every year in the US, more than 2.8 million people acquire a bacterial infection, which are mostly resistant to antibiotics that were previously considered effective for common types of bacterial infections [4]. Currently, approximately thirty-five thousand people die each year in the US due to AMR [4]. There are similar figures in Europe [5]. Improved prescribing of antibiotics improves therapeutic outcomes with the minimum emergence of AMR [6,7,8,9]. However, broad-spectrum antibiotics are often prescribed without an indication, adding to AMR [10,11,12,13]. However, this is not always the case [14]. Having said this, owing to the threat of multidrug-resistant hospital-acquired infections (HAIs), and for the coverage of multiple microbes, mostly broad-spectrum should be started as empiric therapy whilst awaiting the findings from culture and sensitivity testing [6,15,16,17,18,19]. Effective antimicrobial therapy depends on the early identification of causative pathogens through culture sensitivity testing and the appropriate selection of antibiotics according to the results of the sensitivity reports [6,20,21]. Such activities will help avoid rising AMR rates exacerbated by the over use of broad-spectrum antibiotics [6,22,23,24,25,26].

Typically though the results of blood cultures are often ignored because the patients show a therapeutic response to empiric therapy; however, this is not always the case [16,17,25]. Against this, antimicrobial stewardship programmes (ASPs) do help improve subsequent antibiotic utilization patterns in hospitals and reduce subsequent AMR rates [11,27,28,29,30,31,32]. This includes reducing post-operative antibiotic administration to reduce surgical site infections [31,32]. ASPs can also encourage the de-escalation of antibiotic therapy to improve their prescribing [33,34]. However, to date, there is limited information regarding the extent to which culture sensitivity reports help physicians in the selection of the most appropriate antibiotic treatment among low and middle-income countries (LMICs) where resources are more limited and there can be issues with funding sensitivity analyses without good reason [17,35,36]. In one study in India, it was concluded that the result of blood culture reports had a limited effect on the narrowing of antibiotics and the underutilization of culture sensitivity reports has previously been observed in England [16,17]. In their recent systematic review of point prevalence surveys (PPS), Saleem et al. (2020) found generally variable undertaking and documentation of sensitivity patterns due to manpower and cultural issues [37]. In addition, Choudhary et al. (2017) found that a change of therapy was only undertaken in 20.9% of positive culture patients. We are aware that there can be challenges with ordering culture reports among hospitals in LMICs with high rates of empiric prescribing [38,39,40]. For instance in Botswana, culture and sensitivity results were rarely ordered in their PPS study [38]. This is a concern as we are aware that the result of culture reports can help with a reduction in the prescribing of antibiotics. A study found a reduction of 22% in consumption following sensitivity analysis [25].

To the best of our knowledge, only one study to date has been conducted in Pakistan to review the impact of culture sensitivity testing on antimicrobial use in Pakistan [41]. This is important as there are appreciable concerns with AMR in Pakistan driven by their excessive use highlighted in the recent national action plan to address AMR [42]. However, there are currently challenges with its implementation [42]. In addition, we were aware through our recent PPS study in the Punjab region of Pakistan, that in over 75% of cases, the rationale for prescribing a particular antibiotic was not documented in the patient’s notes and that 96.2% of antibiotics were prescribed empirically [40]. Consequently, as a starting point we aimed to address this information gap by ascertaining current resistance patterns of bacterial isolates and the subsequent impact of culture sensitivity test on the use of antibiotics alongside the cost of therapy in a tertiary-care hospital of Pakistan. We believe this is the first time researching this combination has been undertaken in Pakistan, and builds on similar activities in other lower middle income countries [34,43]. We believe the findings can guide subsequent utilization of antibiotics in this leading tertiary hospital in Pakistan and beyond.

## 2. Materials and Methods

### 2.1. Study Design and Study Setting

**Figure 1:**
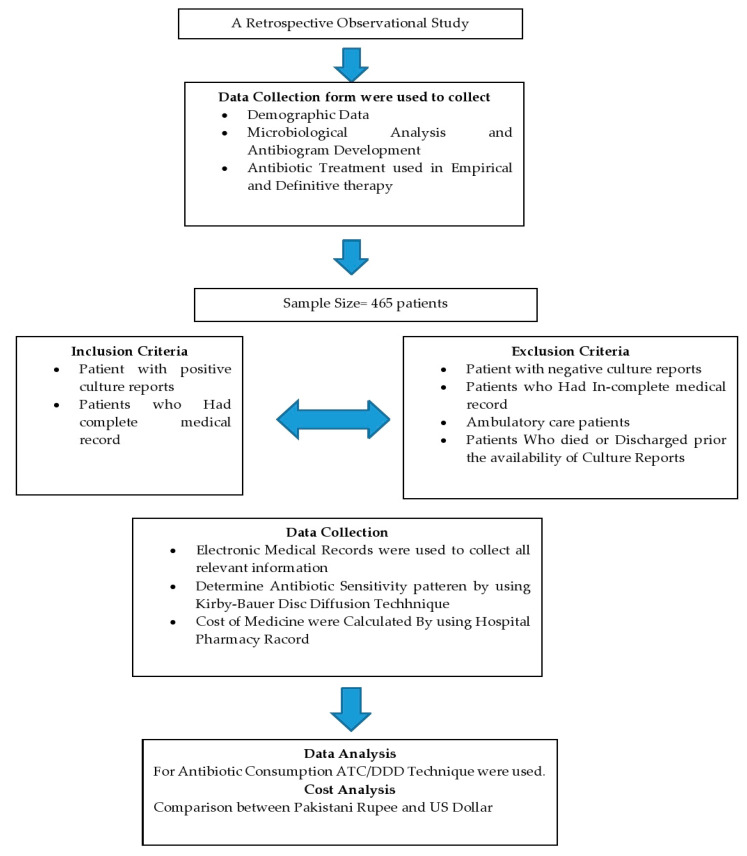
Flowsheet study design.

This retrospective observational study was conducted at Ghurki Trust Teaching Hospital (GTTH). The hospital is a charitable organization in Lahore, Pakistan, with a capacity of 600 beds. The hospital provides health care services from primary to tertiary health care. This hospital has all departments with a particular specialty in orthopedic medicine where the hospital has been awarded the name of the Center of Excellence in Pakistan for arthroplasty and spinal surgery by the Pakistan Orthopedic Association (POA), and POA fellows are being trained regularly in this hospital. Overview of study design is shown in Figure 1.

### 2.2. Study Tool

A standardized paper data collection form was used to collect all information during the study period between May 2018 and December 2018. The data collection form consisted of three principal parts. The first part contained patient demographic data, i.e., the patient’s age, gender, the total length of hospitalization, ward, past surgical history, and the treatment based on any biomarker data. The second part consisted of the type of the sample, causative agent identification, and the sensitivity pattern of antibiotics. The last part consisted of the brand name, generic (INN) name, route, frequency, duration, indication, treatment type, and cost of the antibiotics used for empiric and definitive therapy [25,44].

### 2.3. Definitions

A positive culture report is defined as the presence of one or more microorganisms in the patient collected sample. A polymicrobial culture is defined as the growth of two or more microorganisms in the patient sample. Empiric treatment is defined as antibiotics being started before the result of culture reports are available. Definitive therapy is defined as the treatment started after the availability of culture reports.

### 2.4. Inclusion and Exclusion Criteria

Patients with positive culture reports during the study duration were included in this study. The patients with a negative culture report and ambulatory care patients were excluded from our study because we were principally concerned with in-patient care in this study. Patients who died prior to the index of culture report or were discharged earlier prior to the availability of culture reports were also excluded. Finally, patients with medication records that had irrelevant or incomplete information were also excluded from the study.

### 2.5. Data Collection

Electronic medical records were reviewed to collect demographic data, sample type, causative microbe, and antimicrobial treatment given in empirical and definitive treatment. Culture sensitivity reports were reviewed to collect data on the results of pathogen identification and susceptibility testing. The antibiotic susceptibility pattern of all the bacterial pathogens was determined by Kirby-Bauer Disc Diffusion Technique according to Clinical and Laboratory Standards Institute (CLSI) guidelines [45]. The interpretation of any test was undertaken according to CLSI guidelines as sensitive and resistant. In the case of positive culture reports, patients were observed for their whole length of hospital stay (from the day of positive culture report to the last day of their treatment) to determine the consumption of antibiotics, length of hospital stay, and the cost of the antibiotics prescribed. Susceptibility patterns of pathogens were noted in order to observe the pattern of culture-guided definitive therapy. The cost of medicines was calculated using the hospital’s pharmacy records.

### 2.6. Data Analysis

For antibiotic consumption, Data were analyzed by using the ATC/DDD (Anatomical Therapeutic Chemical & Defined Daily Dose) methodology established by the World Health Organization [46]. ATC/DDD system is an internationally recognized tool for the measurement of drug utilization and is used for comparison purposes at national and international levels [47,48,49,50,51]. In the ATC classification system, drugs are classified into different groups based on the organ system upon which they act, as well as their chemical, pharmacological and therapeutic characteristics. Define Daily Dose (DDD) is a unit of measurement, and it is defined as the assumed average maintenance dose per day for a drug used for its main indication in adults. Only those medicines with an ATC code can have DDD values. The DDD value of drugs is defined by WHO and is updated regularly as new prescribing information becomes available. DDDs of commonly used antibiotics for empiric and definitive treatment are calculated separately [52]. In addition, antibiotic consumption for a certain period of time in hospitals can be calculated by 100 patient admission and for 1000 patient days for comparison purposes [51,53]. This is different from documenting the number of patients in a hospital being prescribed antibiotics as part of a PPS study [37]. The Statistical Process for Social Sciences (SPSS version) program was selected to analyze the data obtained (Descriptive Statistics). Results were presented in the form of frequency, and percentages in the form of tabular and graphical representations.

### 2.7. Cost Analysis

The cost of antibiotics used in empiric and definitive treatment were calculated by calculating the per-day cost of each antibiotic (by taking the current selling price) and then multiplying this by the total number of days patients received that particular antibiotic. The cost savings were calculated by subtracting the cost of definitive therapy from the cost of empiric therapy. We calculated the cost in Pakistani rupees and also in US Dollars for comparison purposes (1 US dollar = 153.75 Pakistani Rupees).

### 2.8. Ethical Approval

Ethics Approval was obtained from the hospital ethics committee (Health Care Ethical Committee) before starting the study (Ref No 5574). The study was performed according to the ethical standards of the hospital and data were collected according to the defined time duration.

## 3. Results

### 3.1. Demographic Characteristics of Selected Patients

A total of 465 patients were identified with positive culture reports. Out of these patients, 299 (64.3%) were men and 166 (35.7%) were women. The majority of the patients were aged between 19–40 years (38.3%). Table 1 depicts the past surgical history of studied patients and it showed that the majority of patients suffered from SSIs (62.8%). The parenteral route of administration was very prevalent (81.2%). Co-morbid conditions presented in the majority of patients, with diabetes mellitus being the most prevalent co-morbidity (21.7%). The majority of the patients were admitted to the orthopedic ward reflecting the fact that GTTH is a POA training hospital. Different samples were taken for microbiological identification and the majority of the samples were taken from pus.

### 3.2. Microbiological Finding of Positive Culture Reports

A total of 497 isolates were detected from the 465 patient samples as 32 patients had polymicrobes. 62.2% of Gram-negative rods were isolated and 37.8% were gram-positive cocci. Table 2 shows that among the 188 isolated gram-positive microorganisms, the most common pathogen was Methicillin-sensitive *S. aureus* in 125 isolates (25.1%). Among the 309 isolated gram-negative microorganisms, the most common pathogen was *E. coli* in 140 (28.1%) isolates.

### 3.3. Sensitivity Pattern of Antibiotics

Antibiotic sensitivity testing showed that *E. coli* was sensitive for more than 75% cases with fosfomycin (100%), colistin (95.8%), polymyxin-b (93.7%), tigecycline (92.8%), amikacin (86%), imipenem (82.5%), chloramphenicol (79.4%) and for meropenem (78%). *Pseudomonas aeruoginosa* showed more than 75% sensitivity to chloramphenicol (100%), polymyxin-b (96%), and colistin (95.8%). *Klebsiella pneumoniae* showed more than 75% sensitivity to meropenem (80%), imipenem (90.3%), and chloramphenicol (94.7%) and 100% to fosfomycin, polymyxin-b, colistin and in tigecycline. *A. baumannii* showed more than 75% sensitivity to polymyxin-b and colistin (96.2%). *Proteus mirabilis* showed more than 75% sensitivity to meropenem (85.1%), piperacillin, and tazobactam (84%) and 100% to cefoperazone + sulbactam and to tigecycline.

*MSSA* showed more than 75% sensitivity to cefoxitin (100%), vancomycin (100%), linezolid (95.7%), tigecycline (95.4%), amikacin (90.1%), minocycline (89.7%), rifampicin (88.5%). co-amoxiclav (80%) clindamycin (77.6%) and gentamicin (75.9%).

Ampicillin and co-amoxiclav showed high levels of resistance in Gram-negative bacteria. Cephalosporins and fluoroquinolones also exhibited high levels of resistance in all Gram-negative bacteria and even in *MSSA*. All the *A. baumannii* isolates showed high levels of resistance to all antibiotics even to carbapenems. Some resistant strains of linezolid were also observed in *MSSA*, which is a concern for the future. The overall situation regarding AMR is of considerable concern (Table 3) that urgently needs to be addressed.

### 3.4. DDDs for Commonly Used Antibiotics, for 100 Patient Admission and for 1000 Patient Days

The high use of parenteral co-amoxiclav (Number of DDDs = 149.66), cefoperazone + sulbactam (Number of DDD = 1100.25), ceftriaxone (Number of DDD = 519), amikacin (Number of DDD = 830.59), vancomycin (Number of DDD = 256.15) and parenteral metronidazole (Number of DDD = 375.11) were observed in empiric treatment (Table 4). In comparison with definitive treatment, the most common antibiotics prescribed were piperacillin + tazobactam (Number of DDD = 372.53), meropenem (Number of DDD = 260.31), oral levofloxacin (Number of DDD = 194), oral clindamycin (Number of DDD = 187.5) and oral linezolid (Number of DDD = 407.68). Compared with empiric treatment, antibiotic consumption decreased by 13.8% with definitive treatment.

### 3.5. Cost Analysis

The average cost per day of antibiotics used in definitive treatment was 8.2% less than seen with empiric treatment. The total average duration of patient hospitalization was 20 days, and the average duration after the availability of culture sensitivity reports was 8 days. This showed that the culture sensitivity reports helped in the reduction of the total length of hospitalization and ultimately reduced the overall costs related to the treatment of patients in this hospital. We calculated the cost in Pakistani rupees and also in US dollars for comparison purposes as shown in Table 5.

### 3.6. Adjustment of Empirical Therapy after the Availability of Culture Sensitivity Reports

**Figure 2:**
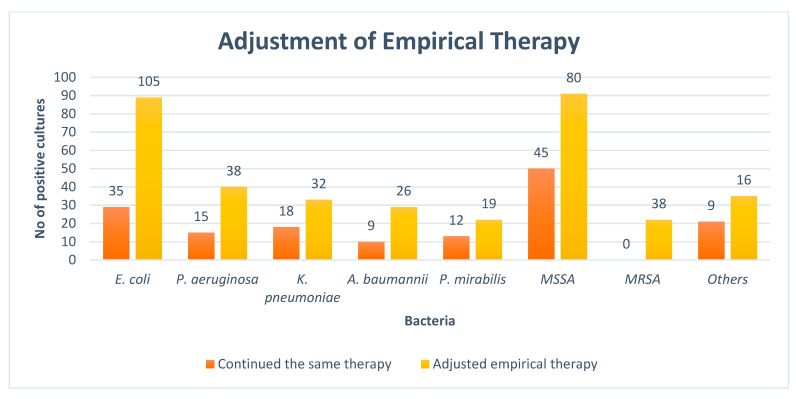
Adjustment of empirical therapy. NB: *E. coli = Escherichia coli*, *P. aeruginosa = Pseudomonas aeruginosa*, *K. pneumoniae = Klebsiella pneumoniae*, *A. baumannii = Acinetobacter baumannii*, *P. mirabilis = Proteus mirabilis*, *MSSA = Methicillin-sensitive staphylococcus aureus*, *MRSA = Methicillin-resistant staphylococcus aureus,* Others *= Streptococcus pyogenes*, *Staphylococcus epidermidis*, *Enterococcus faecalis*, and *Staphylococcus saprophyticus*.

At the time of the culture report, empiric therapy was given to 93.1% of the observed patients. The antibiotics administered were subsequently adjusted in 220 (71.1%) cases of Gram-negative pathogens followed by 134 (71.2%) in Gram-positive pathogens after the result of the culture sensitivity test. The rates of the adjustment of antibiotics after the availability of culture reports are given in Figure 2.

## 4. Discussion

We believe this is the first study undertaken in Pakistan that comprehensively looks at the impact of culture and sensitivity testing on subsequent antibiotic prescribing and costs versus continued empiric prescribing. This is increasingly important in Pakistan given rising AMR rates and continued activities to try and address this [42].

In our current study, the most common bacteria isolated from the patient’s sample was gram-positive (*MSSA*), with similar results seen in other studies [16,17,54]. However, in other studies, Gram-negative bacteria appear more common than Gram-positive bacteria [35]. This may well reflect different bacteria seen in different hospital care settings with different patient populations. In our study, most of the patients were suffering from bone diseases, and mostly *MSSA* or gram-positive bacteria were involved in osteomyelitis and bone infections [55]. The second most common bacteria found in our study was *E. coli*, which is similar to another study conducted in a tertiary care hospital in India [16] but different from another study conducted in England [17].

A high proportion of patients in our study were prescribed antibiotics before the availability of culture results, which is similar to other studies [56]. However, different from a recent study in South Africa where 83% of antibiotics were modified following sensitivity reports [57]. This prescribing behavior may be due to a desire to prevent patients from severe infections without waiting for sensitivity reports. However, empiric therapy can subsequently be adjusted according to the culture sensitivity reports to help reduce unnecessary prescribing of particularly broad-spectrum antibiotics and associated costs.

The most commonly used antibiotics for empiric treatment in our study were cefoperazone + sulbactam followed by amikacin. This is in contrast with the high use of piperacillin + tazobactam and meropenem as empiric therapy in other studies [16], as well as the high use of ceftriaxone in the recent point prevalence study in Pakistan [40]. This may be due to the ready availability of cefoperazone + sulbactam in GTTH and less resistance currently against associated bacteria. The high prescribing of amikacin may be due to the high prevalence of *E. coli*, with amikacin having good therapeutic coverage against *E. coli*. However, we are aware that the main reasons for selecting antibiotics for empiric treatment needs to be investigated further to improve future antimicrobial use in this and other hospitals in Pakistan.

For definitive treatment, the most common antibiotic prescribed was linezolid, which contrasts with meropenem in another published study in lower-middle-income countries [16]. This may well be due to the ready availability of this antibiotic and less resistance against potential pathogens. However, again this needs further investigation.

In this study, the highest use of antibiotics was seen in ICU, which was similar to the findings seen in Kenya and Switzerland [39,58], with high rates of antibiotic use in ICUs also seen in the recent PPS study in Pakistan [40]. However, different from a recent PPS in South Africa, which found no appreciable difference in antibiotic prescribing by ward type [51]. Overall, the extensive use of broad-spectrum antibiotics in definitive treatment could be explained by high bacterial resistance rates [59]. AMR may be reversed if the over-use of antibiotics, especially broad-spectrum antibiotics in the WHO ‘Watch’ category is decreased [60].

Encouragingly in our study, empiric therapy was adjusted in 68.9% of the patients. This is greater than the study conducted by Chuodhary et al. (2017)-47.27% [16]; however, lower than the study conducted by Berild et al. (2005)-88% [25]. Adjustments were mostly undertaken in gram-negative bacteria compared with gram-positive bacteria, which is similar to the study by Berild et.al [25]. However, we currently do not know why the results of culture reports were often ignored in this hospital. This may be because physicians mostly rely on the apparent clinical situation of the patient rather than the result of culture reports [35]. However, this will be investigated further in future studies as this is a concern. Once ascertained, we will also seek to ensure that the findings from any culture sensitivity testing are rapidly conveyed to the prescribing physicians to minimize prescribing of briad-spectrum antibiotics.

In our study, it was observed that no national or international guidelines were available in the hospital to guide empiric therapy [61]. This needs to be urgently addressed as the high use of unnecessary antibiotics increases AMR rates [62], with adherence to guidance known to improve future antibiotic use [8,63]. Our results suggest there is an urgent need to develop guidelines as well as instigate ASPs in this hospital to reduce unnecessary prescribing of antibiotics [64]. This also applies to other hospitals in Pakistan to help improve future antimicrobial prescribing in hospitals [65] and should be part of the Pakistan National Action Plan going forward [66].

Another major concern in our study was the high use of parenteral antibiotics (81.8%), which is similar to the findings of James et al. (2015) [67] as well as the Global and Botswana PPS studies [38,68]. However, appreciably higher than a recent PPS study in South Africa (64.3%) [51]. High use of parenteral antibiotics may well reflect physicians’ and patients’ views that the IV route is more effective compared with the oral route [69]. Whilst the parenteral route is typically preferable in critically ill patients including ICU patients where they are often unable to take oral medicines, or in life-threatening indications where no oral equivalent is available [69], the oral route is generally preferable where possible to reduce the risk of cannula related infection and thrombophlebitis, reduce the length of stay in hospital and ultimately the overall cost of treatment [69,70,71,72,73,74]. Many antibiotics are now available for switching as they have more than 90% bioavailability in their oral form. These include linezolid, fluoroquinolones, doxycycline, metronidazole, and rifampicin [69].

Encouragingly, the consumption of antibiotics was 13.8% lower in the definitive treatment group as compared with empirical treatment, similar to the findings of Berild et al. (2006) [25]. Encouragingly as well, the cost of antibiotics used in definitive therapy was 8.2% lower versus the empiric therapy group, again similar to the findings of Berild et al. [25]. The true cost savings may well be higher as the early availability of culture sensitivity reports decreased the length of stay, which would ultimately decrease the overall cost of patient care, similar to the findings of Stevenson et al. (2012) [75].

Our study also showed that continuous surveillance of susceptibility testing is necessary for cost-effective customization of empiric antibiotic therapy. As a result, providing guidance to other hospitals in Pakistan and beyond.

It was also encouraging to see that only 3.3% of the antibiotics were prescribed by their brand name as opposed to generic medicines, in line with WHO guidance. This is because generic medicines tend to be considerably less expensive than brand-name medicines [76,77,78]. This high rate is encouraged by the WHO in their suggested quality targets [79,80]. In addition, considerably less than seen in Bangladesh (78%), Islamabad (23%), Karachi (12.26%), and Hyderabad (12%) [81,82,83]. Higher rates can be achieved through more instigating stringent bioequivalent studies given some of the concerns in Pakistan [84,85].

In this study, the most common gram-negative microbe was *E. coli*. Encouragingly, antibiotic sensitivity testing showed that *E. coli* had maximum (more than 75%) sensitivity to fosfomycin (100%), colistin (95.8%), polymyxin-b (93.7%), tigecycline & nitrofurantoin (92.8%), amikacin (86%), imipenem (82.5%), erythromycin (80%), chloramphenicol (79.4%) and meropenem (78%). This compares with Nirangan et al. (2014), who showed a maximum sensitivity of imipenem (98.9%), amikacin (82.6%), nitrofurantoin (82.1%), and Piperacillin + tazobactam (78.2%) for *E. coli* [86]. In addition, Gales et al. (2012) showed maximum sensitivity to aztreonam (78.8%), cefoxitin (85.8%), ceftriaxone (75.3%), ceftazidime (82.1%), cefepime (84.1%), gentamicin (81.2%), tobramycin (75.7%) and more than 90% in piperacillin+tazobactam (90.5%), imipenem (99.6%), meropenem (99.9%), amikacin (98.6%) and colistin (99.8%) for *E. coli* [87]. This may reflect different hospital types, locations, and study years. However, we will be monitoring sensitivity patterns closely in this hospital in the future to help further guide appropriate antibiotic choices.

The most common gram-positive microbe was *MSSA* with antibiotic sensitivity testing showing that *MSSA* had maximum (more than 75%) sensitivity to cefoxitin (100%), vancomycin (100%), linezolid (95.7%), tigecycline (95.4%), chloramphenicol (94.1%), amikacin (90.1%), minocycline (89.7%), rifampicin (88.5%), clindamycin (77.6%) and gentamicin (75.9%). This compares with Mir et al. (2016), who showed a maximum sensitivity of vancomycin, linezolid, rifampicin, chloramphenicol, clindamycin, amikacin, fusidic acid, and gentamicin in the case of *MSSA* at 100%, 98.9%, 95.7%, 94.7%, 86.2%, 84%, 83%, and 76.6% respectively [88]. Our findings are a concern, especially with some strains of *MSSA* showing resistance to linezolid, which may well be due to the overuse of linezolid in Pakistan. We will now be investigating this further given the concerns with rising resistance rates to this antibiotic.

We are aware of a number of limitations with this study. Firstly, no standard antibiotic guidelines were available for the selection of appropriate empiric and definitive therapy; consequently, it was impossible to determine physician adherence to hospital guidelines. This is a concern as adherence to hospital guidance is increasingly recognized as a key quality indicator, especially for ASPs [38,51,68]. Nonetheless, this was the first step to identifying the prescribing patterns of physicians after the availability of culture sensitivity reports. Secondly, our study was an observational study; consequently, we did not interfere with physician prescribing trends in the selection of antibiotic treatment. However, in the future, the impact of the involvement of a pharmacist or other key stakeholders actively involved in guiding antibiotic selection in hospitals will be investigated. Lastly, to the best of our present knowledge, no such research has been undertaken before in Pakistan with a special focus on culture sensitivity reports; consequently, we were unable to compare our results with any existing studies in Pakistan. Despite these limitations, we believe our findings are robust with this study highlighting the impact of culture sensitivity reports on antibiotic use as well as the significance of culture-guided therapy on definitive versus empiric treatment. In addition, this study highlighted the need for antibiotic guidelines for the selection of appropriate antibiotics in empiric and definite treatment helped by the instigation of ASPs. The recent availability of the AWaRe book with suggested treatments for an appreciable number of infectious diseases seen across sectors will help here [89].

## 5. Conclusions

Overall, culture sensitivity reports helped to reduce antibiotic utilization in this hospital, decreasing hospital stays and reducing costs. However, there were concerns about the high rates of antimicrobial resistance patterns observed as well as high rates of IV administration. Consequently, there is an urgent need for the implementation of ASPs and the development of hospital antibiotic guidelines in this hospital, and potentially wider in Pakistan. As a result, seek to reduce unnecessary prescribing of broad-spectrum antibiotics and improve the rationality of antibiotics through culture sensitivity reports. We will be monitoring these developments in the future.

## Figures and Tables

**Table 1:** Demographic data of patients.

Parameters	Number	Percentage
Gender		
Male	299	64.3%
Female	166	35.7%
**Age group division**		
Less than 18 year	95	20.4%
19–40 years	178	38.3%
41–60 years	140	30.1%
61–85 years	52	11.2%
**Route of administration**		
Parenteral	1676	81.2%
Oral	389	18.8%
**Prescribing Trend**		
Brand Prescribing	1987/2060	96.7%
Generic prescribing	73/2060	3.3%
**Past surgical history**		
Yes	292	62.8%
No	173	37.2%
**Treatment Based On biomarkers**		
ESR	67	14.4%
CRP	66	14.1%
**Length of Hospitalization**		
Average LOH before the availability of Culture reports (days)	12	
Average LOH after the availability of Culture reports (days)	8	
**Ward**		
Orthopedic	314	67.5%
ICU	69	14.9%
Medical	34	7.3%
Surgical	28	6%
Paeds	13	2.8%
Gynaecology	7	1.5%
**Nature of the sample**		
Pus	272	58.5%
Urine	86	18.5%
Tissue	50	10.8%
Sample from catheterization	21	4.5%
Bone	18	4%
Blood	10	2.1%
Others	8	1.6%
**Co-morbidities**		
Diabetes	101	21.7%
Hypertension	61	13.11%
Hepatitis C	54	11.6%
Tuberculosis	14	3%
Ischemic Heart Disease	13	2.7%
Quadriplegic	12	2.5%

NB: ESR = Erythrocyte sedimentation rate; CRP = C-reactive protein; LOH = Length of Hospitalization; ICU = Intensive care unit; Others included High vaginal swab (3 specimens), Sputum (3 specimens), and Cerebrospinal fluid (2 specimens).

**Table 2:** Microbiological findings of positive culture reports.

Organism	Number	Percentage
**Gram-positive bacteria**		
Methicillin-sensitive *Staphylococcus aureus*	125	25.1%
Methicillin-resistant *Staphylococcus aureus*	38	7.6%
*Streptococcus pyogenes*	12	2.4%
*Staphylococcus epidermidis*	8	1.6%
*Enterococcus faecalis*	4	0.8%
*Staphylococcus saprophyticus*	1	0.2%
**Gram-negative bacteria**		
*Escherichia coli*	140	28.1%
*Pseudomonas aeruginosa*	53	10.6%
*Klebsiella pneumonia*	50	10.%
*Acinetobacter baumannii*	35	7%
*Proteus mirabilis*	31	6.2%

**Table 3:** Sensitivity pattern of antibiotics.

Antibiotics	*Escherichia coli*	*Pseudomonas aeruginosa*	*Klebsiella pneumoniae*	*Acinetobacter baumannii*	*Proteus mirabilis*	Methicillin Sensitive *staphylococcus aureus*
Ampicillin	6/94(6.3%)	NA	NA	NA	NA	6/29 (20.6%)
Co –amoxiclav	11/74 (14%)	NA	9/34 (36.4%)	NA	3/20 (15%)	8/10 (80%)
Piperacillin + Tazobactam	62/94 (65.9%)	22/31(70%)	19/27 (70.3%)	9/27 (33.3%)	21/25 (84%)	NA
Penicillins	NA	NA	NA	NA	NA	24/113(21.1%)
Cefepime	8/37 (21.6%)	8/22 (36.3%)	5/12 (41.6%)	3/20 (15%)	5/11 (45.4%)	NA
Cefixime	2/44 (4.5%)	NA	1/9 (11.1%)	NA	NA	NA
Cefuroxime	4/75 (5.3%)	NA	2/28 (7.1%)	NA	2/21 (9.5%)	NA
Ceftriaxone	8/84 (9.5%)	NA	7/31 (22.5%)	NA	5/21 (23.8%)	NA
Cefazolin	NA	NA	NA	NA	NA	21/35 (60%)
Cefoxitin	NA	NA	NA	NA	NA	107/107(100%)
Ceftazidime	10/23 (43%)	13/28 (36.4%)	9/20 (45%)	NA	4/7 (57.1%)	NA
Cefoperazone + Sulbactam	12/21 (57.1%)	NA	3/6 (50%)	4/14 (28.5%)	6/6 (100%)	NA
Meropenem	78/100 (78%)	18/34 (52.9%)	28/38 (80%)	9/32 (28.1%)	23/27 (85.1%)	NA
Imipenem	71/86 (82.5%)	16/26 (61.5%)	28/31 (90.3%)	7/18 (38.8%)	13/22 (59%)	NA
Ertapenem	44/60 (73.3%)	NA	9/16 (56.2%)	NA	11/15 (73.3%)	NA
Ciprofloxacin	15/64 (23.4%)	19/32 (5.9%)	11/26 (42.3%)	1/14 (7.1%)	10/21 (47.6%)	45/99(44.4%)
Norfloxacin	9/39 (23%)	2/9 (22.2%)	1/5 (20%)	NA	NA	21/54 (46.2%)
Levofloxacin	21/69 (30.4%)	12/26 (46.1%)	9/24 (37.5%)	10/22 (45.4%)	8/17 (47%)	19/27 (70.3%)
Erythromycin	NA	NA	NA	NA	NA	84/125 (67.2%)
Clarithromycin	NA	NA	NA	NA	NA	12/22 (54.5%)
Amikacin	80/93 (86%)	26/35 (74.2%)	28/40 (70%)	9/26 (34.6%)	15/27 (55.5%)	101/112 (90.1%)
Gentamicin	47/82 (55.2%)	14/30 (46.6%)	16/31 (51.6%)	6/24 (25%)	24/78 (43.5%)	82/108 (75.9%)
Tobramycin	22/44 (50%)	15/25 (60%)	3/9 (33.3%)	7/22 (31.8%)	2/8(25%)	37/54 (68.5%)
Tetracycline	13/72 (18%)	NA	8/22 (36.3%)	2/15 (13.3%)	3/12 (25%)	41/83 (49.3%)
Minocycline	16/36 (44.4%)	NA	4/9 (44.4%)	20/29 (68.9%)	2/8 (25%)	35/39 (89.7%)
Tigecycline	39/42 (92.8%)	NA	8/8 (100%)	18/25 (72%)	6/6 (100%)	42/45 (95.4%)
Co-trimoxazole	9/65 (13.8%)	1/9 (11.1%)	4/12 (33.3%)	NA	2/16 (12.5%)	17/79 (21.5%)
Clindamycin	3/6 (50%)	NA	NA	NA	NA	97/125(77.6%)
Chloramphenicol	31/39 (79.4%)	9/9 (100%)	18/19 (94.7%)	4/10 (40%)	6/16 (37.5%)	81/86 (94.1%)
Nalidixic Acid	7/39 (17.9%)	1/7 (14.2%)	NA	NA	NA	NA
Fosfomycin	44/44 (100%)	NA	3/3 (100%)	NA	NA	NA
Polymyxin-b	45/48 (93.7%)	24/25 (96%)	14/14 (100%)	26/27 (96.2%)	2/7 (28.5%)	NA
Colistin	46/48 (95.8%)	23/24 (95.8%)	13/13 (100%)	27/28 (96.2%)	0/9 (100%)	NA
Vancomycin	NA	NA	NA	NA	NA	121/121 (100%)
Teicoplanin	NA	NA	NA	NA	NA	44/66 (66.6%)
Linezolid	NA	NA	NA	NA	NA	114/119 (95.7%)
Rifampicin	NA	NA	NA	NA	NA	54/61 (88.5%)

NB: NA = Not available.

**Table 4:** Calculation of DDDs for commonly used antibiotics, for 100 patient admissions, for 1000 patient days.

Antibiotics (ATC Code)	DDDs		For 100 Patient Admission		For 1000 Patient Days	
	Empirical Treatment	Definitive Treatment	Empirical Treatment	Definitive Treatment	Empirical Treatment	Definitive Treatment
Cefoperazone + sulbactam (J01DD62)	1100.25	349.87	4.49	1.42	12.83	4.08
Amikacin (J01GB06)	830.59	298.56	3.39	1.21	9.69	3.48
Piperacillin + tazobactam (J01CR05)	246.82	372.53	1.00	1.52	2.87	4.34
Ceftriaxone (J01DD04)	519	135	2.11	0.55	6.05	1.57
Meropenem (J01DH02)	174.86	260.31	0.71	1.06	2.04	3.03
Oral Ciprofloxacin (J01MA02)	359	344.75	1.46	1.40	4.18	4.02
Parenteral Ciprofloxacin (J01MA02)	138	116.63	0.56	0.47	1.60	1.36
Parenteral Metronidazole (J01XD01)	375.11	147.10	1.53	0.60	4.37	1.71
Oral Metronidazole (P01AB01)	12.4	23.4	0.05	0.09	0.14	0.27
Vancomycin (J01XA01)	256.15	137.10	1.04	0.55	2.98	1.59
Parenteral Amoxicillin and clavulanic acid (J01CR02)	149.66	37.66	0.61	0.15	1.74	0.43
Oral Amoxicillin and clavulanic acid (J01CR02)	53.7	32	0.21	0.13	0.62	0.37
Parenteral Moxifloxacin (J01MA14)	132	70	0.53	0.28	1.53	0.81
Oral Moxifloxacin (J01MA14)	20	10	0.08	0.04	0.23	0.11
Parenteral Levofloxacin (J01MA12)	66.5	106.83	0.27	0.43	0.77	1.24
Oral Levofloxacin (J01MA12)	96	194	0.39	0.79	1.11	2.26
Tigecycline (J01AA12)	29	140	0.11	0.57	0.33	1.63
Parenteral Clindamycin (J01FF01)	95.33	80.33	0.38	0.32	1.11	0.93
Oral Clindamycin (J01FF01)	17.75	187.5	0.07	0.76	0.20	2.18
Parenteral Linezolid (J01XX08)	56.05	129.62	0.22	0.52	0.65	1.51
Oral Linezolid (J01XX08)	60.53	407.68	0.24	1.66	0.70	4.75
Colistin (J01XB01)	0.88	23	0.003	0.09	0.01	0.26

NB: DDD = Define Daily Dose; ATC = Anatomical Therapeutic Chemical Classification System of the World Health Organization; For 100 Patient Admission = Number of DDDs per year/Number of patients warded for that particular year × 100, For 1000 Patient Days = Number of DDD per year/Total Number of patient days for that particular year × 100.

**Table 5:** Cost of antibiotics.

Variables	Before the Availability of Culture Reports (A)	After the Availability of Culture Reports (B)	Difference in Cost (A–B)
Per Day Average Cost of Antibiotics (In Pakistani Rupees)	PKR = 9225	PKR = 8467	PKR = 758 (8.2%)
Per Day Average Cost of Antibiotics (In US Dollar)	60$	55$	5$

PKR = Pakistan rupees.

## Data Availability

The data presented in this study are available on request from the corresponding author.
